# *In situ* Synthesis of Au-Induced Hierarchical Nanofibers/Nanoflakes Structured BiFeO_3_ Homojunction Photocatalyst With Enhanced Photocatalytic Activity

**DOI:** 10.3389/fchem.2018.00649

**Published:** 2019-01-04

**Authors:** Yan'an Li, Jiao Li, Long Chen, Haibin Sun, Hua Zhang, Hong Guo, Liu Feng

**Affiliations:** ^1^School of Materials Science and Engineering, Shandong University of Technology, Zibo, China; ^2^Analysis & Testing Center, Shandong University of Technology, Zibo, China

**Keywords:** bismuth ferrite, homojunction, SPR effect, defects, photodegradation

## Abstract

In order to further improve the photocatalytic performance of BiFeO_3_ (BFO), novel Au-induced hierarchical nanofibers/nanoflakes structured BiFeO_3_ homojunctions (Au_*x*_-BFO, *x* = 0, 0. 6, 1.2, 1.8, 2.4 wt%) were *in situ* synthesized through a simple reduction method with assist of sodium citrate under the analogous hydrothermal environment. The effect of loading amount of Au nanoparticles (NPs) on the physicochemical properties and photocatalytic activity was investigated in detail. The Au_1.2_-BFO NFs sample show the best photocatalytic activity (85.76%), much higher than that for pure BFO samples (49.49%), mainly due to the hierarchical nanofibers/nanoflakes structured homojunction, the surface plasmon resonance (SPR) effect of Au NPs, as well as the presence of defects (Fe^2+^/Fe^3+^ pairs and oxygen vacancy). Furthermore, the possible formation mechanism of the unique homojunction and the enhanced photocatalytic mechanism for the degradation of methylene blue (MB) dye are proposed. It is proven that holes (h^+^) play the decisive role in the photocatalytic process. The present work provides a fascinating way to synthesize efficient homojunctions for the degradation of organic pollutes.

## Introduction

Nowadays, energy crisis and environmental deterioration issues are severely detrimental for economic development and human health (Wang et al., 2013). Semiconductor photocatalysis as an efficient and green technology can convert solar energy into chemical energy to dispose of these issues (Wang et al., 2013; Wang H. et al., 2017). In order to fully exploit the solar energy, it is necessary to explore visible light responsive photocatalysts with excellent photocatalytic activities (Zhang et al., 2017).

Among the variety of visible-light-driven semiconductor materials, BiFeO_3_ (BFO) has attracted a great deal of attention due to its narrow band gap (2.2~2.5 eV), good chemical stability and low cost (Wang et al., 2013; Niu et al., 2015). In addition, the room temperature multiferroic property makes it easily recovered from the treated water to avoid the secondary pollution (Zhang et al., 2017). However, the rapid recombination rate of photogenerated electron-hole pairs and low quantum yield limit its practical application (Huo et al., 2011; Srivastav and Gajbhiye, 2012; Niu et al., 2015). Therefore, much efforts have been made to improve its photocatalytic performance, such as element doping (Wang et al., 2012; Ahmada et al., 2017; Irfan et al., 2017; Yang et al., 2018), morphology control (Mohan and Subramanian, 2013; Zhang Q. et al., 2016), noble metal deposition (Li S. et al., 2013; Zhang et al., 2015), and semiconductor coupling (Li Z. et al., 2013; Humayun et al., 2016).

Compared to that of bulk materials, the reduced radial dimension and the extremely large surface-to-volume ratio of one-dimension (1D) nanofibrous structure can promote the rapid transfer of photogenerated charge carriers to the surface, and thus enhance the separation efficiency (Li S. et al., 2013). Furthermore, the perpendicular transport direction of photogenerated electrons and holes can further effectively inhibit the recombination of photogenerated electron-hole pairs (Mohan and Subramanian, 2013). Therefore, the synthesis of BFO sample with nanofibrous structure is in favor of enhancing the photocatalytic activity.

Noble metal (e.g., Ag, Au, Pt, etc.) deposition is also an efficient way to enhance the photocatalytic activity (Li et al., 2015; Niu et al., 2015; Zhang et al., 2015). Among them, Au nanoparticles (NPs) exhibit excellent a chemical stability and a characteristic absorption peak in the visible wavelength range due to the strong surface plasmon resonance (SPR) effect (Li S. et al., 2013; Li et al., 2015). The deposited Au NPs can not only act as electron-trapping centers to reduce the recombination rate of photogenerated electron-hole pairs, but also can act as light harvesters enhancing the light absorbing ability and as catalytic sites for the photocatalytic reaction (Lin et al., 2017; Chiu et al., 2018). Significantly, the formed Schottky junctions between Au NPs and BFO nanofibers (NFs) can adjust the interfacial band structure and thus promote the separation and transfer of photogenerated charge carriers.

In comparison with heterojunction built with different semiconductors. Besides the band alignment of two semiconductors, the creation of heterojunctions is also dependent on other properties of semiconductor, such as electron affinity and work function (Ye et al., 2017). The homojunction is constructed by the same semiconductor materials with different crystal phases, exposing facets or semiconductor types, *etc*., which can avoid the disadvantages of heterojunction fabrication (Huang et al., 2017). The enhanced photocatalytic activity of heterojunctions or homojunctions is mainly dependent on the space charge accumulation or depletion at the interfaces of two phases (Ye et al., 2017). The homojunction structure can also introduce an internal field to effectively facilitate surface charge separation, retard the recombination of photogenerated carriers, and thus remarkably improve the photocatalytic performance. However, there are few reports on the fabrication of BFO homojunction, especially for the aspect of *in situ* synthesis.

In this work, an distinctive Au NPs deposited BFO homojunction, in which BFO nanoflakes are assembled on the surface of BFO NFs, was *in situ* synthesized through a simple reduction method under the analogous hydrothermal environment. The effect of loading amount of Au NPs on the physicochemical properties and photocatalytic activity was investigated in detail. Furthermore, it proposed a possible formation mechanism of the unique homojunction and the enhanced photocatalytic mechanism for the degradation of methylene blue (MB) dye over Au_*x*_-BFO NFs under simulated solar light irradiation.

## Experimental

### Preparation of Au_x_-BFO NFs

BFO NFs (Au_*x*_-BFO NFs, *x* = 0 wt%) were prepared *via* a sol-gel method combined with an electrospinning technique. All the chemical reagents used for the synthesis were analytical grade. Bismuth nitrate (Bi(NO_3_)_3_·5H_2_O) and ferric nitrate (Fe(NO_3_)_3_·9H_2_O) with the Bi: Fe molar ratio of 1.11:1 (to make up for the loss of Bi ions during the calcination process) were dispersed in methylglycol by ultrasonic to form a homogeneous solution. Then the viscosity and pH value of the solution were adjusted by 2.5 mL of glacial acetic acid, called solution A. g of poly(vinylpyrrolidone) (PVP, M*w* = 1,300,000) was added to the mixed solvent of N'N-dimethylformamide (DMF) and ethanol with the volume ratio of 2:1, called solution B. Solution B was added dropwise to solution A with constantly stirring for 24 h to form the pre-spinning solution which has a pH of 2.0. The pre-spinning solution was loaded into a plastic syringe connected with a stainless needle and then fixed onto the electrospinning system. An optimized high voltage of 15 kV was applied to the needle with the flow rate of 0.4 mL/h. The distance was 20 cm from the needle to the rotating drum collector. The as-spun NFs were dried at 60°C for 4 h, calcined at 350°C for 30 min with a heating rate of 2°C/min, and then calcined at 600°C for 120 min with a heating rate of 5°C/min in air.

Au NPs deposited BFO NFs (Au_*x*_-BFO NFs, *x* = 0.6, 1.2, 1.8, 2.4 wt%) were synthesized by a simple reduction method with sodium citrate. In a typical procedure, 57.38 mg of BFO NFs were first dispersed in 50 mL of deionized water, followed by adding a certain amount of HAuCl_4_ (3 mmol/L) and sodium citrate (0.04 mol/L). Then the suspension was kept stirring at 150°C for 30 min in oil bath. After cooled to room temperature naturally, the obtained products were centrifuged, washed with deionized water and absolute ethanol for several times, and then dried at 80°C for 24 h. The loading amount of Au NPs on BFO NFs were 0.6, 1.2, 1.8, and 2.4 wt% (mass ratio of Au to BFO), which were defined as Au_0.6_-BFO NFs, Au_1.2_-BFO NFs, Au_1.8_-BFO NFs, and Au_2.4_-BFO NFs, respectively.

### Characterization

The phase structures were identified by X-ray diffraction (XRD; D8 Advance, Bruker AXS, German) with Cu Kα radiation (λ = 1.5418 Å) at 40 kV and 50 mA in a 2θ ranging from 20 to 70°. The morphologies were observed by field emission scanning electron microscope (FE-SEM; Sirion 200, FEI, USA). The transmission electron microscopy (TEM) and high resolution TEM (HRTEM) were performed at a field emission transmission electron microscope (TEM; Tecnai G2 F20 S-TWIN, FEI, USA) with an accelerating voltage of 200 kV. UV-vis diffuse reflectance spectra (DRS) were measured at room temperature by UV-Vis spectrometer (UV-Vis; UV-3600plus, Shimadzu, Japan) with the wavelength range of 350–800 nm using BaSO_4_ as a reflectance. The measurements of magnetic properties were carried out by a superconducting quantum interference device magnetometer (FM, MPMS XL-7, Qunatum Design, USA). The element chemical states were characterized by X-ray photoelectron spectrometer (XPS; PHI-5300, PHI, USA) with an Al Kα X-ray radiation. The photoluminescence (PL) spectra were measured on the fluorescence spectrophotometer (PL; Hitachi F-4600, Hitachi, Japan) with the excitation wavelength of 230 nm and a 150 W xenon lamp as the light source.

### Photocatalytic Activity Evaluation

Twenty five milligram of Au_*x*_-BFO NFs were added to 30 mL of 10 mg/L MB solution with ultrasound for 10 min. After stirring in the dark for 30 min to reach the adsorption-desorption equilibrium, the suspension system was exposed to simulated solar light irradiation (CHF-XM-500W xenon lamp, Beijing Perfactlight Company, China). During the photocatalytic reaction process, the suspension was taken out at every 30 min interval and centrifuged to remove the photocatalyst. The MB concentration was measured by a UV-vis spectrophotometer at the wavelength of 666 nm.

## Results and Discussion

### XRD Analysis

**Figure 1:**
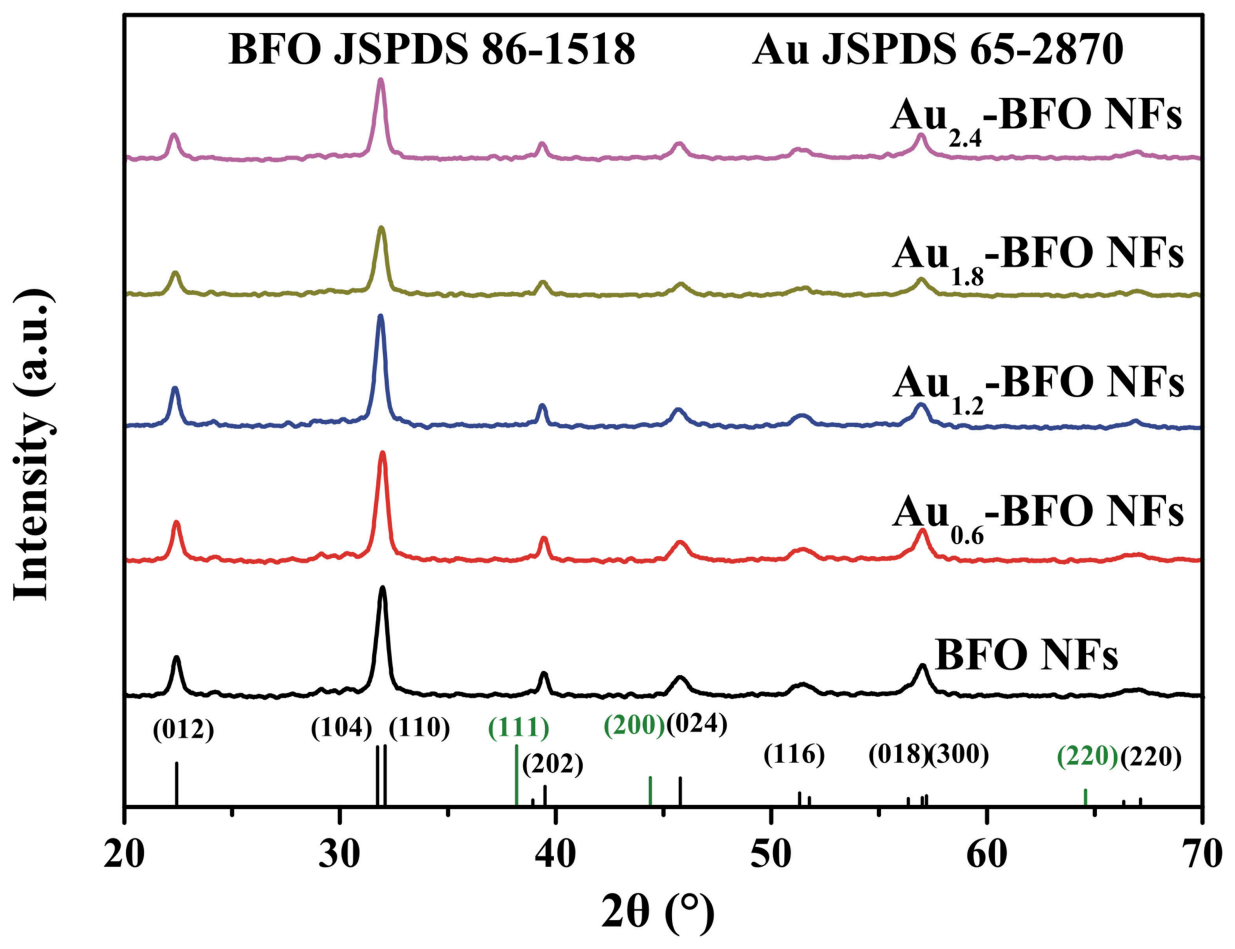
XRD patterns of Au_*x*_-BFO NFs (*x* = 0, 0.6, 1.2, 1.8, 2.4 wt%).

The XRD patterns of Au_*x*_-BFO NFs (*x* = 0, 0.6, 1.2, 1.8, 2.4 wt%) are shown in Figure 1. All the diffraction peaks of pure BFO NFs can be indexed to the rhombohedral perovskite structure with *R*3*m* space group of the BiFeO_3_ phase (JCPDS card no. 86–1518) (Chen et al., 2017). The strong diffraction peaks also imply the good crystallinity. In addition, the crystalline structures of BFO NFs are not changed after depositing Au NPs.

### Microstructures

**Figure 2:**
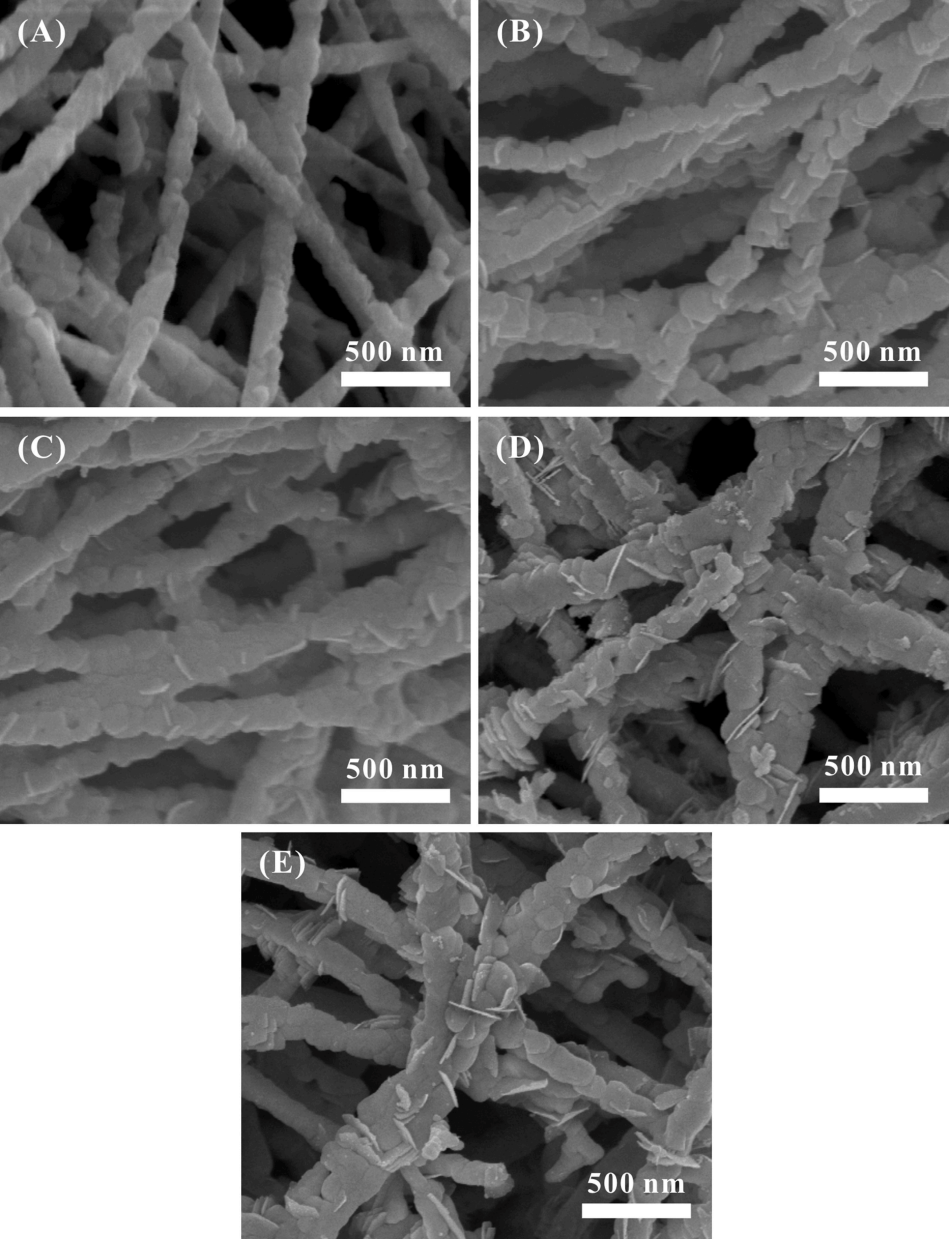
SEM images of Au_*x*_-BFO NFs: **(A)** *x* = 0 wt%, **(B)** *x* = 0.6 wt%, **(C)** *x* = 1.2 wt%, **(D)** *x* = 1.8 wt%, **(E)** *x* = 2.4 wt%.

Figure 2 shows the SEM images of Au_*x*_-BFO NFs (*x* = 0, 0.6, 1.2, 1.8, 2.4 wt%). It can be seen from Figure 2A that the pure BFO NFs exhibit an uniform nanofibrous structure with compactly packed and continuous nanoparticles. The diameter of BFO NFs is about 100–200 nm. As shown in Figures 2B–E, there is no obvious change of the nanofibrous structure for BFO NFs with Au NPs deposition, but some thin nanoflakes are embedded in the surface of nanofibers, and the number of nanoflakes increases with the increase of the loading amount of Au NPs.

**Figure 3:**
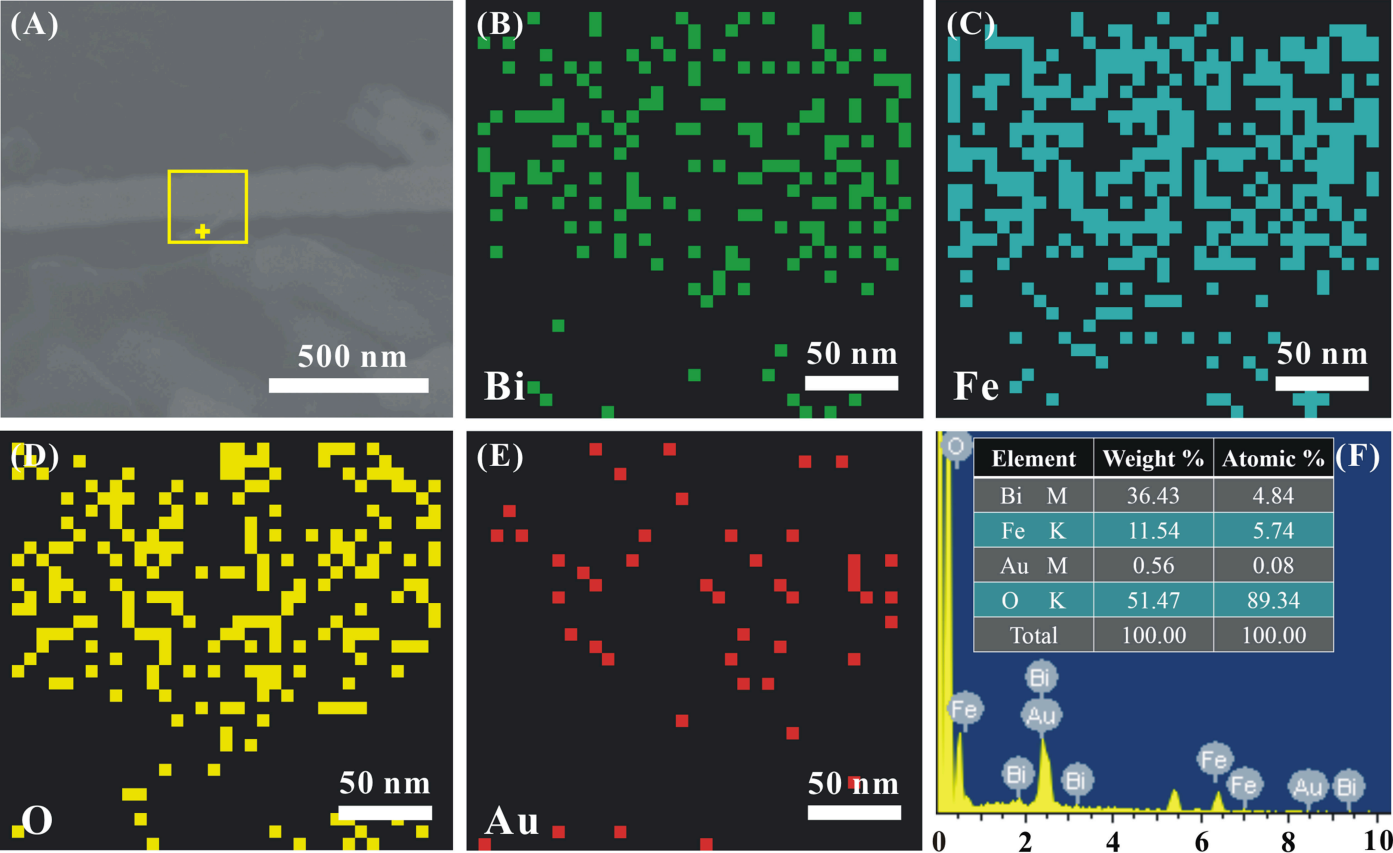
**(A)** The SEM image of a single representative Au_1.2_-BFO NFs, the corresponding elemental mappings of **(B)** Bi, **(C)** Fe, **(D)** O, **(E)** Au elements, and **(F)** EDS pattern.

The EDS mapping is performed to clarify the chemical composition and element distribution in Au_1.2_-BFO NFs, as shown in Figure 3. It can be clearly seen that Bi, Fe, and O elements are uniformly distributed throughout the single nanofiber (Figures 3A–D). Au NPs are dispersed well on BFO NFs, instead of agglomerating together (Figure 3E). More importantly, The EDS result (Figure 3F) recorded at the “+” position in Figure 3A shows that the main elements of the nanoflake are Bi, Fe, Au, and O. It is worthy to note that the atomic ratio of Bi/Fe is almost 1:1, nearly corresponding to the composition of BFO. The trace amount of Au shows that Au NPs are also deposited on the nanoflake.

**Figure 4:**
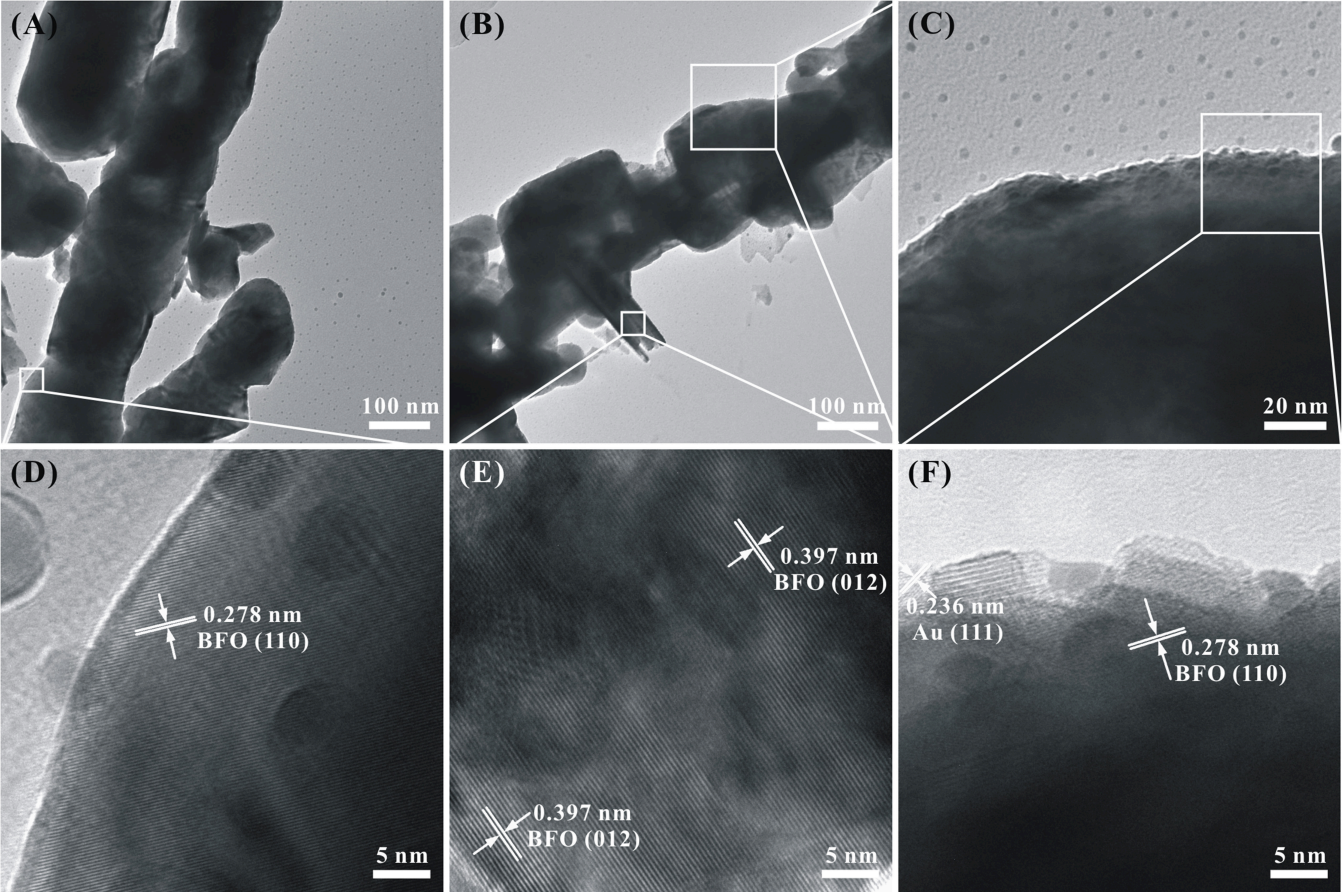
TEM and HRTEM images of **(A,D)** pure BFO NFs and **(B,C,E,F)** Au_1.2_-BFO NFs.

In order to further illustrate the morphology and microstructure of pure BFO NFs and Au_1.2_-BFO NFs, TEM technology is used. As shown in Figure 4A, the pure BFO sample is nanofibrous structure compactly packed with nanoparticles. The HRTEM image in Figure 4D is collected from the square area in Figure 4A. The interplanar spacing of 0.278 nm corresponds to the (110) lattice plane of the rhombohedral perovskite BFO structure. Figure 4B shows the TEM image of Au_1.2_-BFO NFs. It can be seen that the morphology is still the nanofibous structure. However, the surface of the nanofiber becomes rougher and the composed nanoparticles appear to be more prominent after depositing the Au NPs on the surface of BFO NFs. Furthermore, new nanoflake-like structures are formed and embedded in the BFO NFs. In order to confirm the structure of nanoflakes, the HRTEM is used, and the result of which is shown in Figure 4E. The interplanar spacing of 0.397 nm is ascribed to the (012) lattice plane of the rhombohedral perovskite BFO structure, further implying that the new formed nanoflake-like structure is indeed BFO phase. Therefore, homojunctions will be formed between BFO NFs and BFO nanoflakes. From Figure 4B, there is no obvious Au NPs deposited on the BFO surface. In order to clearly observe the deposited Au NPs, the magnified TEM image is shown in Figure 4C. It can be seen that Au NPs with the size of about 5 nm are indeed deposited uniformly on the BFO surface. The according HRTEM image is shown in Figure 4F. The interplanar spacing of 0.236 nm matches well with the (111) lattice plane of the cubic Au structure (Wang P. et al., 2017).

**Figure 5:**
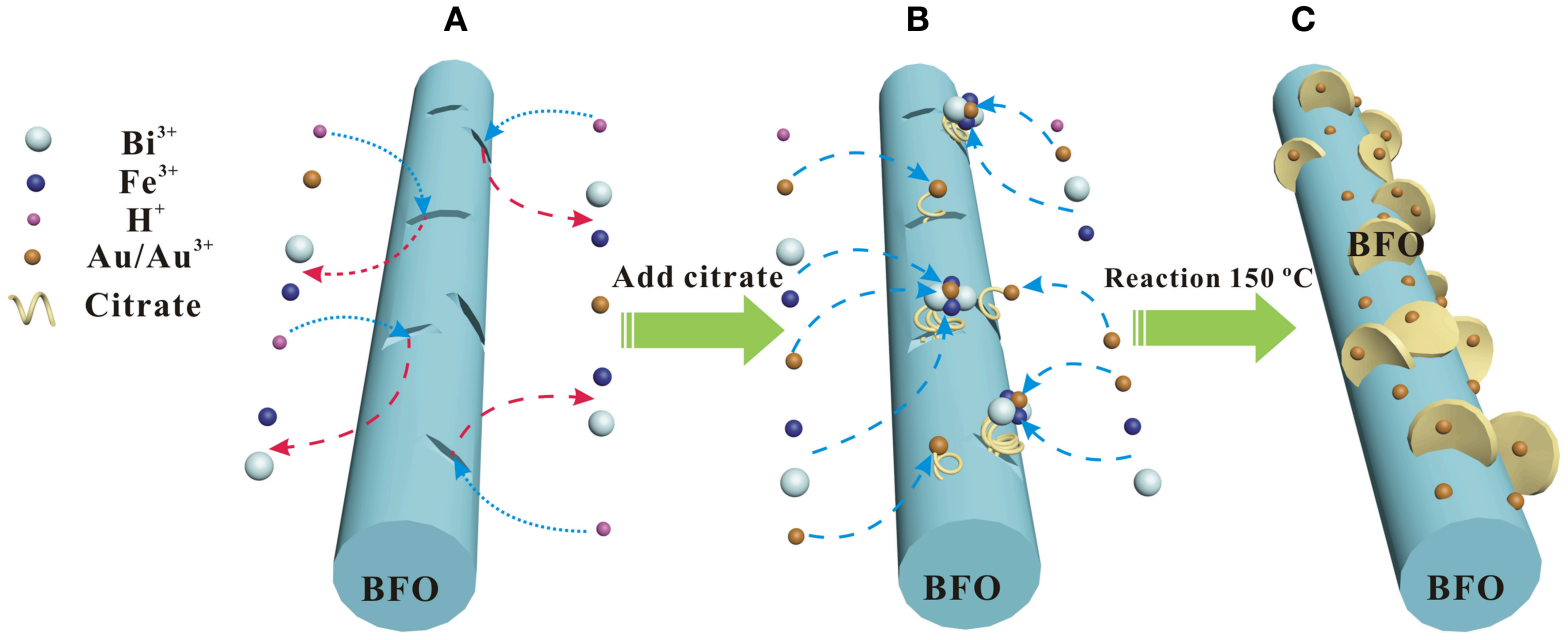
Scheme of the possible formation mechanism of Au NPs deposited hierarchical nanofibers/nanoflakes structured BiFeO3 homojunction: **(A)** the BiFeO3 dissolution process, **(B)** metal-chelate complexs formation process, **(C)** Au NPs and nanoflake-like BiFeO3 structure formation process.

The possible formation mechanism of the Au NPs deposited hierarchical nanofibers/nanoflakes structured BFO homojunction is proposed in Figure 5. Firstly, the HAuCl_4_ is decomposed to AuCl_3_ and HCl in the aqueous solution. BiFeO_3_, being regarded as the product of a combination of Bi_2_O_3_ and Fe_2_O_3_, is soluble in the weak acidic environment (Sakar et al., 2013). Therefore, the generated HCl can decompose the BiFeO_3_ to generate Bi^3+^ and Fe^3+^ ions in the solution (Figure 5A). In particular, the decomposition reaction prefers to occur at the defects on the surface of BFO NFs where the higher surface energy have. The defects may be generated during the oxidation decomposition of organic material (PVP) under the high calcination temperature. Secondly, the citrate present in the solution can act as a chelating agent to react with Bi^3+^, Fe^3+^, and Au^3+^ ions to form stable metal-chelate complexes (Figure 5B). In addition, due to the presence of defects, the citrate can anchor on the surface of BFO NFs (Mudunkotuwa and Grassian, 2010). Finally, the uniform Au NPs and nanoflake-like BFO structure are formed on the surface of BFO NFs under the effect of the citric acid in the analogous hydrothermal environment (Figure 5C). With the increase of the HAuCl_4_ content (the increase of the loading amount of Au NPs), more Bi^3+^ and Fe^3+^ ions pass from the defects on the surface of BFO NFs to the solution, and more nanoflake-like BFO structure are formed.

### XPS Analysis

**Figure 6:**
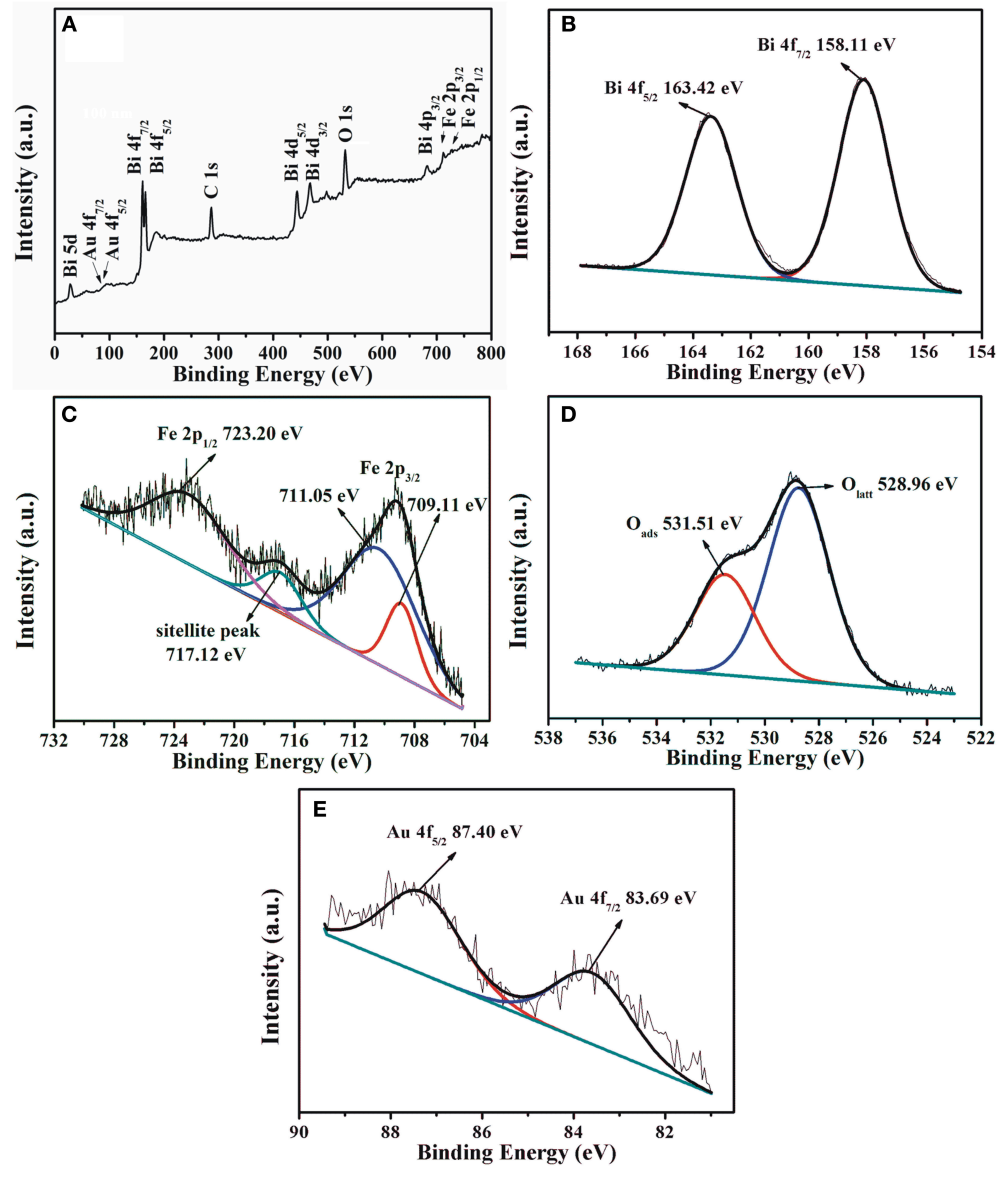
XPS patterns of the Au_1.2_-BFO NFs: **(A)** overall XPS survey, **(B)** Bi 4f, **(C)** Fe 2p, **(D)** O 1s, and **(E)** Au 4f.

The chemical state and elemental composition of Au_1.2_-BFO NFs are analyzed by XPS technology. According to the overall XPS survey (Figure 6A), only Bi, Fe, O, Au, and C elements can be detected in the sample. The peak of C 1s located at 284.5 eV is used for calibration (Dou et al., 2015; Chen et al., 2016). As shown in Figure 6B, the two peaks centered at around 158.11 eV and 163.42 eV can be assigned to the binding energy of Bi 4f_7/2_ and Bi 4f_5/2_, respectively. It is confirmed that the bismuth species in Au_1.2_-BFO NFs is the oxidation state of +3. From the high resolution XPS spectra of Fe 2p in Figure 6C, the three peaks located at 710.79, 717.12, and 723.20 eV can be ascribed to the Fe 2p_3/2_, satellite, and Fe 2p_1/2_ peaks, respectively. In addition, the Fe 2p_3/2_ peak can be fitted into two peaks located at 711.05 and 709.11 eV, which are attributed to Fe^3+^ and Fe^2+^ species, respectively (Wu et al., 2016). The presence of Fe^2+^ species may come from the reduction of Fe^3+^ species by citric acid under high temperature environment. In order to meet the requirement of charge equilibrium, oxygen vacancy should be formed accompanied by the Fe^2+^ species. The O 1s curve in Figure 6D can be splitted into two peaks with Lorentzian-Gaussian function. The peaks at 531.51 and 528.96 eV can be ascribed to the surface adsorbed oxygen (O_ads_) and lattice oxygen (O_latt_) species, respectively (Bharathkumar et al., 2016; Chen et al., 2016). The O_ads_ species may originate from the –OH groups bonded to the metal cations in the oxygen defcient region (Guo et al., 2016; Sun et al., 2017), which can be converted to oxygen-related free radicals to participate in the photocatalytic reaction. Moreover, the more O_ads_ species usually mean the more oxygen vacancies it possesses, and also the better photocatalytic activity. From Figure 6E, the binding energies of 83.69 eV and 87.40 eV are ascribed to the Au 4f_7/2_ and Au 4f_5/2_ peaks, respectively, indicating that the gold is present in the form of metallic gold (Au^0^) in the Au_1.2_-BFO NFs. This can be ascribed to the reduction by citric acid under high temperature environment. Therefore, the metallic gold have been successfully deposited on the surface of BFO NFs. Whereas, the binding energies of Au 4f in the Au_1.2_-BFO NFs are 0.3 eV below the standard binding energy of a metallic gold foil (Jovic et al., 2013), indicating the existence of electron transfer from BFO NFs to Au NPs for the different Fermi levels (Li S. et al., 2013). In addition, the loading amount of Au NPs is measured to be 1.14%, almost equal to the theoretical value of 1.2%.

### Ferromagnetism Analysis

**Figure 7:**
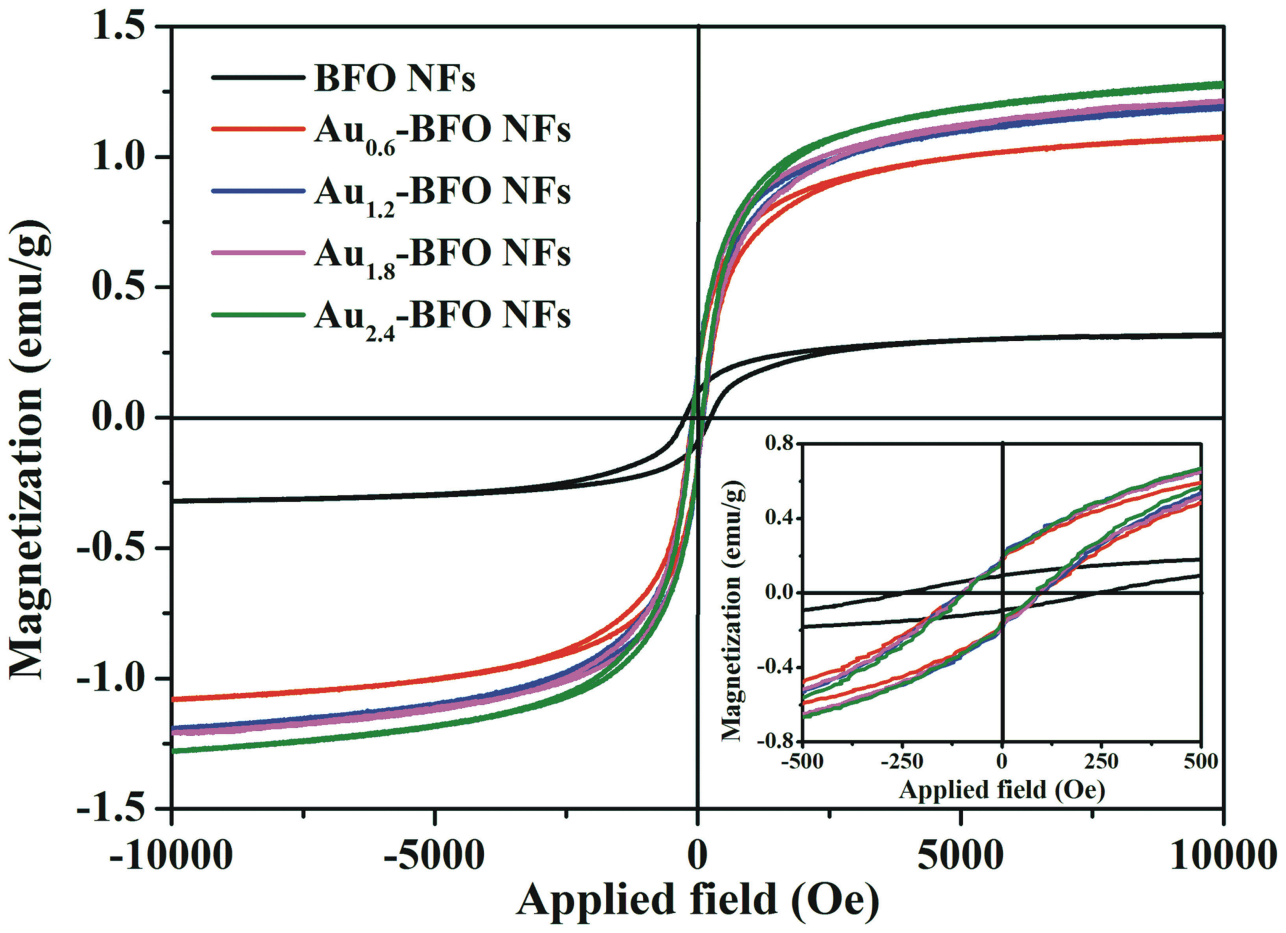
*M–H* hysteresis loops of Au_*x*_-BFO NFs (*x* = 0, 0.6, 1.2, 1.8, 2.4 wt%) measured at room-temperature; the inset shows the partially magnified curves.

In order to investigate the effect of Au on the magnetic property of Au_*x*_-BFO NFs, the room-temperature magnetic hysteresis (*M-H*) loops are shown in Figure 7. It can be seen that all BFO NFs exhibit the weak-ferromagnetic (FM) characters, which is due to the suppressed cycloidal spin structure and the coexistence of Fe^3+^ and Fe^2+^, thereby resulting in the FM spins in the system (Xu et al., 2014; Modak et al., 2016). The pure BFO NFs show the saturation magnetization (*Ms*) value of 0.32 emu/g. When Au NPs are deposited on the surface of BFO NFs, the *Ms* value is enhanced with the increase in the loading amount of Au NPs. The *Ms* values are 1.07, 1.19, 1.22, and 1.27 emu/g for BFO NFs with the loading amount of Au NPs of *x* = 0.6, 1.2, 1.8, and 2.4 wt%, respectively. The gradually enhanced magnetic properties of Au_*x*_-BFO NFs could be attributed to the new formed nanoflake-like structures, where the confined dimensions (15 nm) facilitate the enhancement of FM properties (Chauhan et al., 2012). In addition, the rather large magnetic moment of Au NPs explains the enhanced magnetic properties with increase in loading amount of Au NPs (Nealon et al., 2012). From the inset of Figure 7, an obvious off-center displacement in magnetic field axis is shown in the *M–H* curves. This can be ascribed to the exchange bias (EB) effect of antiferromagnet (AFM) - FM ordering in BFO NFs, where the AFM-core/FM-shell-like structure can be formed in the well-defined 1D structure (Sakar et al., 2016). Compared to that of the pure BFO NFs, the off-center displacement of Au_*x*_-BFO NFs is relatively less pronounced, which is ascribed to the enhanced FM spins caused by the emergence of nanoflake-like structures.

### Optical Properties

**Figure 8:**
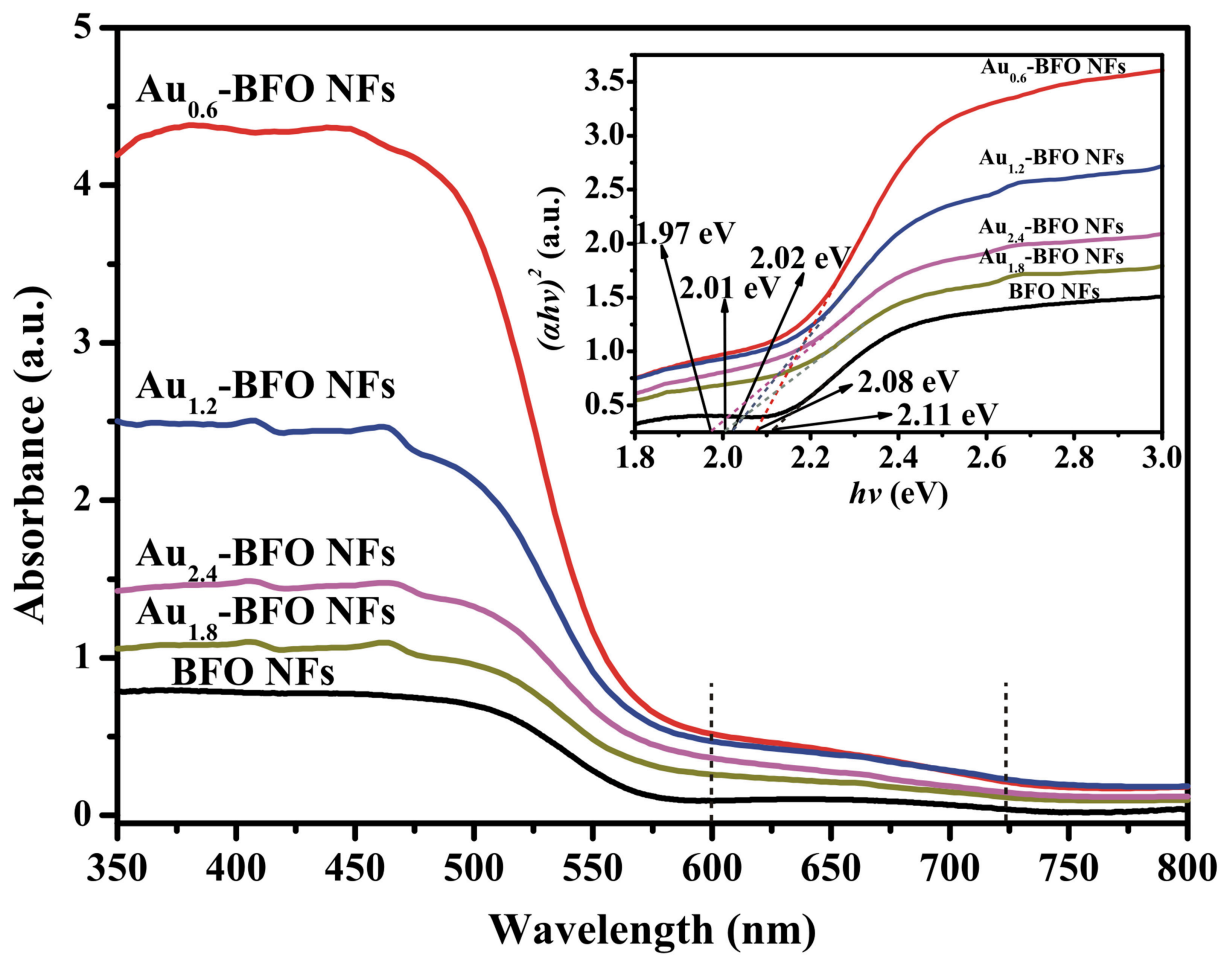
UV–vis DRS spectra of Au_*x*_-BFO NFs (*x* = 0, 0.6, 1.2, 1.8, 2.4 wt%); the inset shows the corresponding band gap energies.

Figure 8 shows the UV-vis DRS spectra of Au_*x*_-BFO NFs (*x* = 0, 0.6, 1.2, 1.8, 2.4 wt%) samples. It can be seen that the pure BFO NFs exhibit excellent absorption in both UV and visible light region. The absorption edge located at 575 nm is due to the bandgap transition. When the Au NPs are deposited on the surface of BFO NFs, the optical properties are obviously enhanced. In addition, the Au_*x*_-BFO NFs exhibit a weak absorption peak in the range of 600~725 nm, which are attributed to the SPR effect of Au NPs (Villa et al., 2016; Wang P. et al., 2017). The enhanced absorption in visible light is in favor of boosting the photocatalytic activity. Moreover, the band gap energies can be calculated from the plots of the (α*h*ν)^2^ vs. the photon energy (*h*ν) (Xu et al., 2015; Singh et al., 2018), the results of which are shown in the inset of Figure 8. The band gap energies are calculated to be 2.11, 2.08, 2.02, 2.01, and 1.97 eV for the *x* = 0, 0.6, 1.2, 1.8, 2.4 wt%, respectively. It is obvious that the band gap energies decrease slightly with the increase in the loading amount of Au NPs. The possible reason is that the deposited Au NPs can produce some lower energy levels due to the formed Schottky junction at the interface between Au NPs and BFO NFs (Papadas et al., 2015).

### PL Analysis

**Figure 9:**
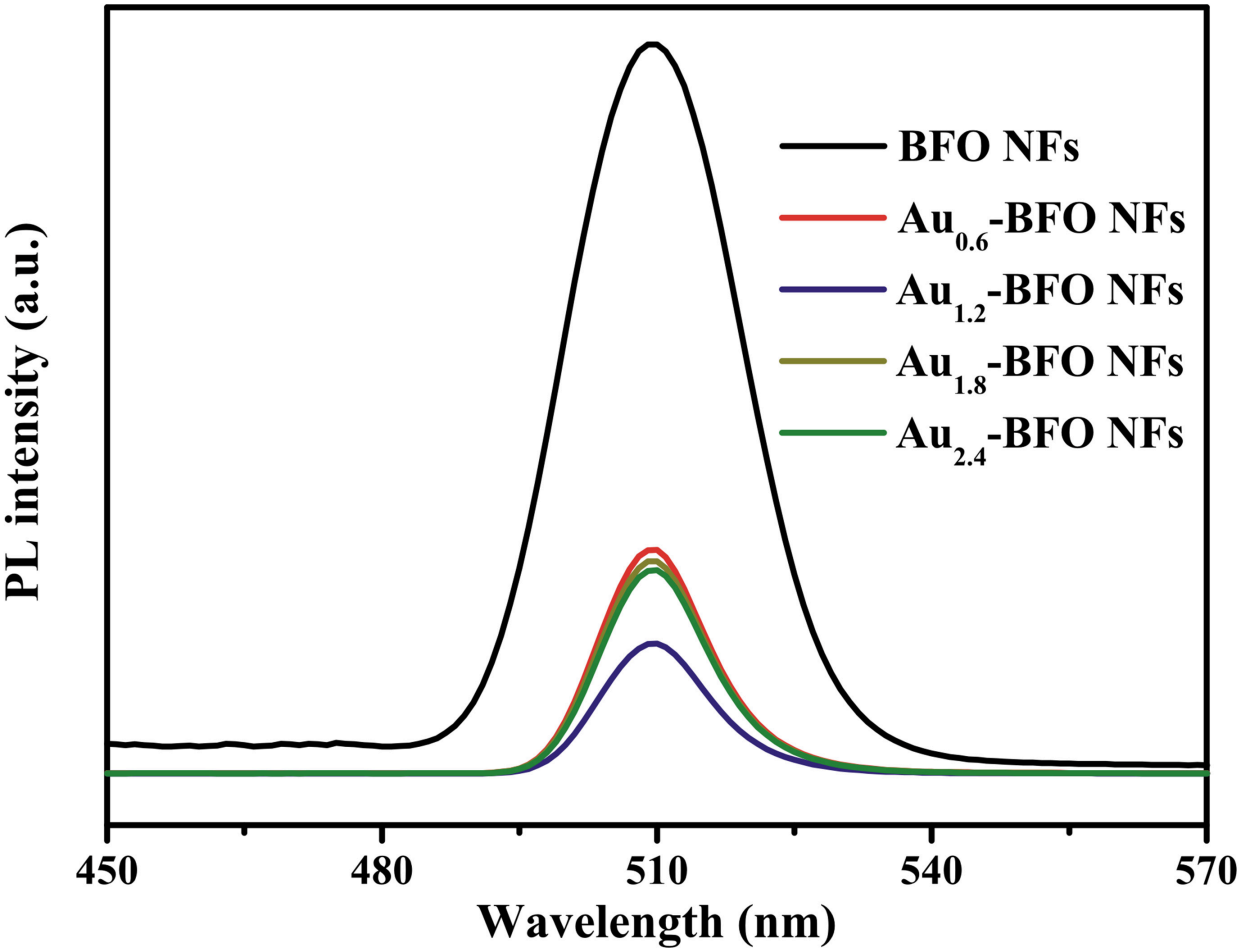
PL spectra of Au_*x*_-BFO NFs (*x* = 0, 0.6, 1.2, 1.8, 2.4 wt%) at the excitation wavelength of 230 nm.

Photoluminescence (PL) spectra are extensively used to study the transfer and separation of photogenerated electron-hole pairs in the semiconductor material. The peak intensity can reflect the capture, migration and transfer efficiency of photogenerated electron-hole pairs. The lower PL intensity usually means the lower recombination rate of photogenerated electron-hole pairs, and also the higher photocatalytic activity (Tang et al., 2018). It can be seen from Figure 9 that the PL intensity decreases obviously once the Au NPs are loaded on the surface of BFO NFs. Significantly, the PL intensity of Au_1.2_-BFO NFs is the lowest. This indicates that the deposition of Au NPs on the surface of BFO NFs can efficiently inhibit the recombination of photogenerated charge carriers, which may be attributed to that the Schottky junction formed between Au NPs and BFO NFs can efficiently promote electron transfer from BFO NFs to Au NPs, and thus decreased the recombination rate of photogenerated charge carriers (Li S. et al., 2013). However, the PL intensity increases when the loading amount of Au NPs is too much. This can be ascribed to the fact that the excess amount of Au NPs can result in an increase of surface defects, which can act as recombination centers for photogenerated charge carriers, thus leading to the increase in the recombination rate. Therefore, it is necessary to deposit an appropriate amount of Au NPs on the surface of BFO NFs to promote the efficient separation of photogenerated electron-hole pairs, thus enhancing the photocatalytic activity.

### Photocatalytic Activities

**Figure 10:**
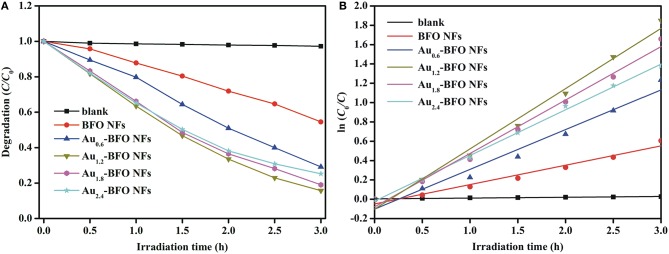
**(A)** Photocatalytic degradation efficiencies of Au_*x*_-BFO NFs (*x* = 0, 0.6, 1.2, 1.8, 2.4 wt%) on the degradation of MB dye under simulated solar light irradiation, and **(B)** kinetic linear simulation curves of MB photocatalytic degradation with Au_*x*_-BFO NFs (*x* = 0, 0.6, 1.2, 1.8, 2.4 wt%).

Figure 10A shows the photocatalytic degradation efficiencies of Au_*x*_-BFO NFs (*x* = 0, 0.6, 1.2, 1.8, 2.4 wt%) on the degradation of MB dye under simulated solar light irradiation. For contrast, the blank experiment without any photocatalyst is carried out under the same conditions. The result shows that the MB dye is hardly degraded under simulated solar light irradiation. The degradation efficiency is only 2.77% after irradiation of 3 h. When the pure BFO sample is added to the reaction system, the degradation efficiency reaches to be 49.49% after irradiation of 3 h. However, the photocatalytic activity is still relatively poor. Once Au NPs are deposited on the surface of BFO NFs, the photocatalytic activities of Au_*x*_-BFO NFs are obviously enhanced. The enhanced activities can be ascribed to the hierarchical nanofibers/nanoflakes structured homojunction, the SPR effect of Au NPs, as well as the presence of defects (Fe^2+^/Fe^3+^ pairs and oxygen vacancy). In addition, the photocatalytic activity is increased and then suppressed with the increase in the loading amount of Au NPs. When the loading amount of Au NPs is 1.2%, the photocatalytic activity is the best with the photocatalytic efficiency of 85.76% after irradiation of 3 h.

To evaluate the reaction kinetics of the degradation of MB, the plot of ln(*C*_0_/*C*) vs. time is plotted in Figure 10B, which shows the kinetics of the degradation process by the five photocatalysts which follow Langmuir-Hinshelwood pseudo first-order kineticsequation, ln(*C*_0_/*C*) = kt, where t is the irradiation time and k is the rate constant (Niu et al., 2015). The observed calculated rate constants k in the presence of blank sample, Au_*x*_-BFO NFs (*x* = 0, 0.6, 1.2, 1.8, 2.4 wt%) are 8.28 × 10^−3^, 200.23 × 10^−3^, 411.46 × 10^−3^, 623.27 × 10^−3^, 552.85 × 10^−3^, and 472.69 × 10^−3^ h^−1^, respectively. which also indicates that the Au_1.2_-BFO NFs sample exhibits the strongest photocatalytic activity among the Au_*x*_-BFO NFs (*x* = 0, 0.6, 1.8, 2.4 wt%) samples.

**Figure 11:**
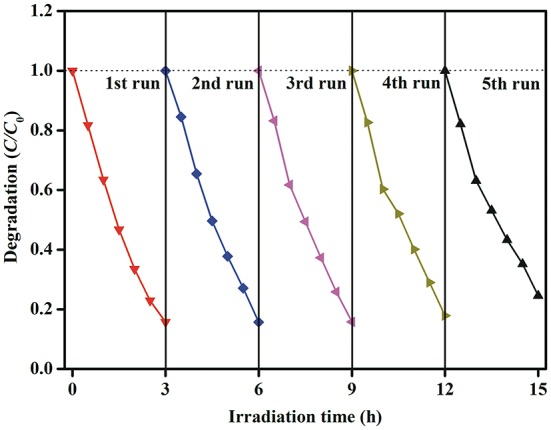
Stability evaluation for the photocatalytic degradation of MB dye in the presence of Au_1.2_-BFO NFs under simulated solar light irradiation.

In order to evaluate the stability of photocatalysts, the recycle experiment is carried out. After each recycle, the photocatalysts are collected by simple filtration and washed with deionized water and absolute ethanol. As shown in Figure 11, there is no obvious loss in photocatalytic activity after five recycle test, indicating that Au_*x*_-BFO NFs photocatalysts have good stability during the photocatalytic degradation of MB dye under simulated solar light irradiation.

**Figure 12:**
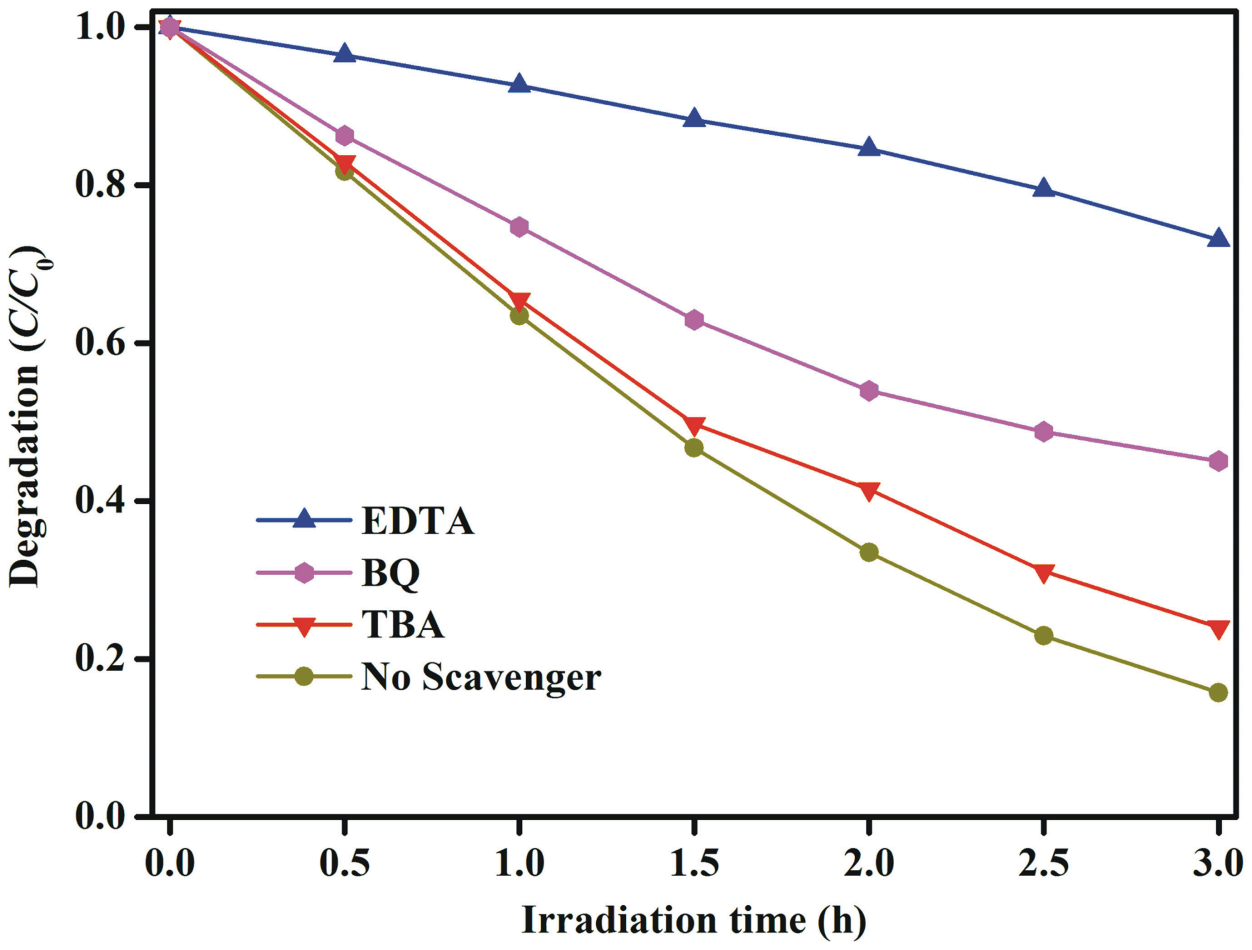
Active species trapping experiments of photocatalytic degradation of MB dye over the Au_1.2_-BFO NFs under simulated solar light irradiation with the addition of EDTA, TBA, and BQ quenchers.

In order to explore the possible reaction pathways during the photocatalytic degradation of MB dye under simulated solar light irradiation, the trapping experiments are used to determine the main active species in this reaction system. Ethylenediaminetetraacetic acid (EDTA), tertbutyl alcohol (TBA) and benzoquinone (BQ) are used as scavengers for holes (h^+^), hydroxyl radicals (•OH), and superoxide radicals (•${\text{O}}_{2}^{-}$), respectively (Chen et al., 2016; Qiao et al., 2017; Ji et al., 2018). As can be seen from Figure 12, when the TBA or BQ is added, a slight reduction in MB degradation efficiency is occurred in comparison to the initial activity without addition of any scavengers, which means that •${\text{O}}_{2}^{-}$ and •OH radicals are not the main active species. However, the photocatalytic activity is severely suppressed when EDTA is added to the reaction system, implying that photogenerated holes are the main active species and play the decisive role in the photocatalytic activity.

### Photocatalytic Mechanism

**Figure 13:**
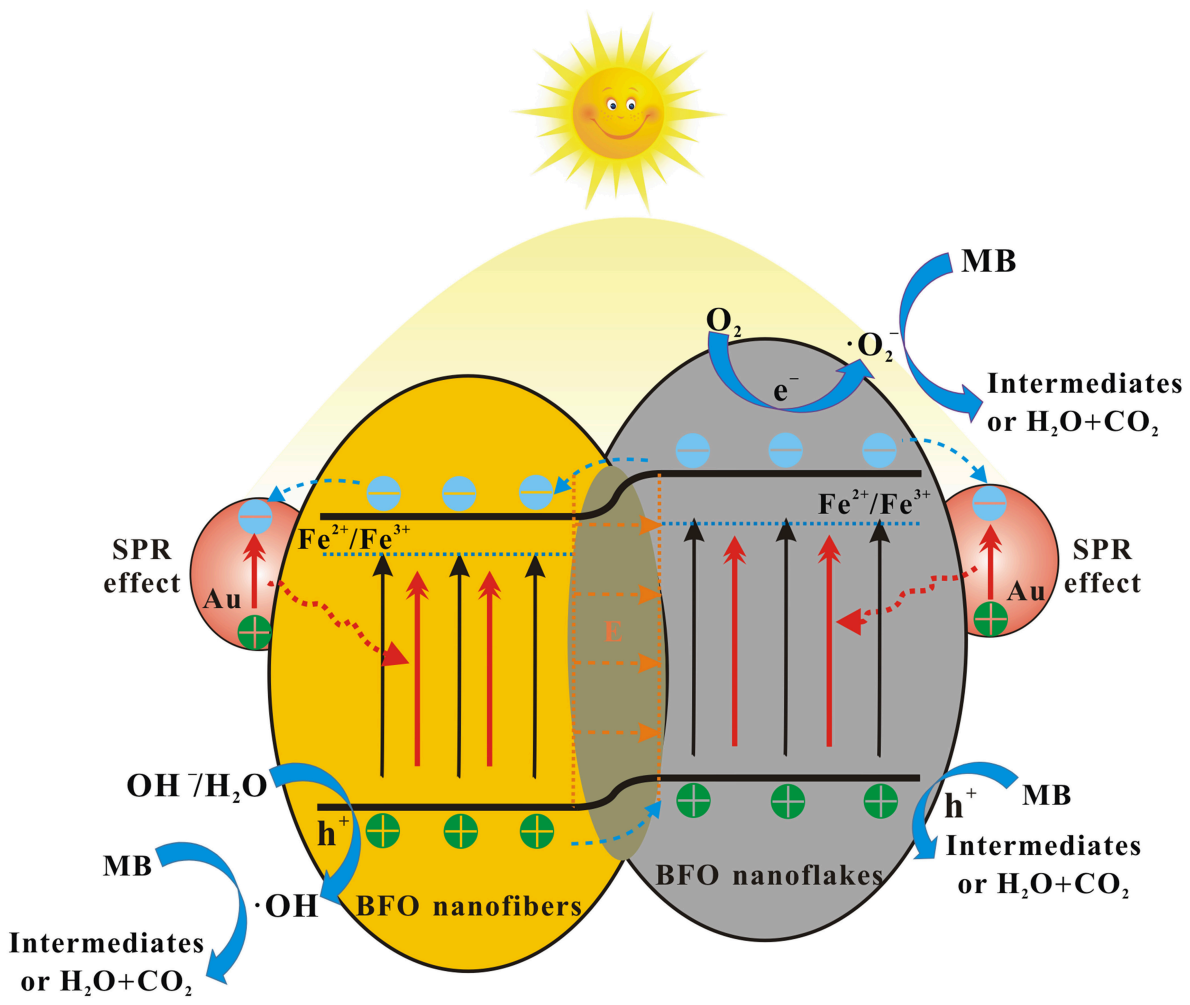
The possible enhanced photocatalytic mechanism for the degradation of MB dye over Au_*x*_-BFO NFs under simulated solar light irradiation.

On the basis of the aforementioned experimental and active species trapping results, the possible enhanced photocatalytic mechanism is illustrated in Figure 13. It can be ascribed to the following aspects: (1) Nanofibers/nanoflakes structured homojunction. It is well-known that nanoflakes with ultrathin structure possess higher valence band (VB) energy than that of nanofibers with larger particle size (Weng et al., 2014). After forming homojunctions between BFO NFs and BFO nanoflakes, the staggered band potentials would introduce a new internal electrical field to provide a driving force to reduce the charge transfer barrier. The formed homojunction can promote the transfer of photogenerated holes (h^+^) from the VB of BFO NFs to the VB of BFO nanoflakes, whereas photogenerated electrons (e^−^) from the conduction band (CB) of BFO nanoflakes to that of BFO NFs. (2) SPR effect of Au NPs. The enhanced local electric field in the near-surface region of BFO induced by the SPR effect of Au NPs results in the further separation of photogenerated electrons and holes. The photogenerated electrons are thermodynamically favorable to transfer from the CB of BFO to Au NPs due to the Fermi energy of Au NPs is more positive than the CB potential of BFO (Li S. et al., 2013). The formed Schottky junction at the interfaces can act as trapping centers for photogenerated electrons, contributing the accumulation of photogenerated electrons at the surface of Au NPs and preventing the recombination of photogenerated electron-hole pairs. (3) Defects. The presence of Fe^2+^/Fe^3+^ pairs located below the CB of BFO can act as electron/hole trapping centers and thus reduce the recombination rate (Zhang et al., 2014; Verma and Kotnala, 2016). In addition, the oxygen vacancy can introduce the defect state energy level and effectively regulate the band structure, thus significantly improve the separation of photogenerated charge carriers (Zhang C. et al., 2016). Under the effect of these aspects, the photocatalytic activities are remarkably enhanced.

During the process of photocatalytic reaction, a few photogenerated electrons in the CB of BFO nanoflakes can react with oxygen molecules adsorbed on the surface to form superoxide radicals (•${\text{O}}_{2}^{-}$). The photogenerated electrons transferred to the Au NPs can react with Fe^2+^/Fe^3+^ pairs and oxygen vacancy defects. Meanwhile, a little part of photogenerated holes in the VB of BFO NFs can oxidize the absorbed OH^−^ and H_2_O to hydroxyl radicals (•OH) (Dong et al., 2016; Hu et al., 2017). Finally, the photogenerated holes transferred the VB of BFO nanoflakes as well as the formed •${\text{O}}_{2}^{-}$ and •OH species can oxidation decomposition of MB dye molecules.

## Conclusions

Novel Au-induced hierarchical nanofibers/nanoflakes structured BFO homojunction (Au_*x*_-BFO, *x* = 0, 0.6, 1.2, 1.8, 2.4 wt%) were *in situ* synthesized by a simple reduction method under the analogous hydrothermal environment. The deposited Au NPs were distributed uniformly on the surface of BFO sample. The formed BFO nanoflakes were vertically assembled on BFO nanofibers to form a hierarchical architecture. The photocatalytic results showed that Au_1.2_-BFO NFs samples exhibited the best photocatalytic activity with the photocatalytic efficiency of 85.76% after irradiation of 3 h. The remarkable enhanced photocatalytic activity could be mainly attributed to the hierarchical nanofibers/nanoflakes structured homojunction, the SPR effect of Au NPs, as well as the presence of defects (Fe^2+^/Fe^3+^ pairs and oxygen vacancy). This work provided a novel method to design efficient homojunctions with multiple functions in solar energy utilization.

## Author Contributions

YL synthesized Au_*x*_-BFO NFs samples and analyzed part of characterizations. JL is the supervisor of this research work. LC and HS helped writing. HZ analyzed Ferromagnetism measurements. HG and LF performed SEM, EDS, and TEM imaging and analyses. All authors have contributed to preparing the review article.

### Conflict of Interest Statement

The authors declare that the research was conducted in the absence of any commercial or financial relationships that could be construed as a potential conflict of interest.
